# Placental transcriptional signatures associated with cerebral white matter damage in the neonate

**DOI:** 10.3389/fnins.2022.1017953

**Published:** 2022-10-28

**Authors:** Carmen Amelia Marable, Kyle Roell, Karl Kuban, T. Michael O’Shea, Rebecca C. Fry

**Affiliations:** ^1^School of Medicine, University of North Carolina at Chapel Hill, Chapel Hill, NC, United States; ^2^Department of Environmental Sciences and Engineering, Gillings School of Global Public Health, University of North Carolina at Chapel Hill, Chapel Hill, NC, United States; ^3^Institute for Environmental Health Solutions, Gilling School of Global Public Health, University of North Carolina at Chapel Hill, Chapel Hill, NC, United States; ^4^Division of Pediatric Neurology, Department of Pediatrics, Boston University Medical Center, Boston, MA, United States; ^5^Department of Pediatrics, School of Medicine, University of North Carolina at Chapel Hill, Chapel Hill, NC, United States

**Keywords:** cerebral white matter damage, transcriptome, mRNA, placenta, placenta-brain-axis, ultrasound

## Abstract

Cerebral white matter is the most common anatomic location of neonatal brain injury in preterm newborns. Factors that predispose preterm newborns to white matter damage are understudied. In relation to studies of the placenta-brain-axis, dysregulated placental gene expression may play a role in preterm brain damage given its implication in programming early life origins of disease, including neurological disorders. There is a critical need to investigate the relationships between the placental transcriptome and white matter damage in the neonate. In a cohort of extremely low gestational age newborns (ELGANs), we aimed to investigate the relationship between the placental transcriptome and white matter damage as assessed by neonatal cranial ultrasound studies (echolucency and/or ventriculomegaly). We hypothesized that genes involved in inflammatory processes would be more highly expressed in placentas of ELGANs who developed ultrasound-defined indicators of white matter damage. Relative to either form of white matter damage, 659 placental genes displayed altered transcriptional profiles. Of these white matter damage-associated genes, largely distinct patterns of gene expression were observed in the study (*n* = 415/659 genes). Specifically, 381 genes were unique to echolucency and 34 genes were unique to ventriculomegaly. Pathways involved in hormone disruption and metabolism were identified among the unique echolucency or ventriculomegaly genes. Interestingly, a common set of 244 genes or 37% of all genes was similarly dysregulated in the placenta relative to both echolucency and ventriculomegaly. For this common set of white matter damage-related genes, pathways involved in inflammation, immune response and apoptosis, were enriched. Among the white matter damage-associated genes are genes known to be involved in Autism Spectrum Disorder (ASD) and endocrine system disorders. These data highlight differential mRNA expression patterning in the placenta and provide insight into potential etiologic factors that may predispose preterm newborns to white matter damage. Future studies will build upon this work to include functional measures of neurodevelopment as well as measures of brain volume later in life.

## Introduction

Each year in the United States alone, more than 380,000 infants are born prematurely (<37 weeks gestational age) (Purisch and Gyamfi-Bannerman, 2017). Globally, this number reaches approximately 15 million infants (Dimes, 2021). Preterm birth predisposes the child to structural brain abnormalities, such as cerebral white matter injury, as well as functional disorders, such as neurodevelopmental impairments (Bhutta et al., 2002). More specifically, infants who are born prematurely have a 1.3 and 2.64 relative risk of developing autism spectrum disorder (ASD) and attention deficit/hyperactivity disorder (ADHD), respectively (Bhutta et al., 2002; Wang et al., 2017). The relative risks for these adverse outcomes are even larger for those who are born extremely prematurely (<28 weeks gestational age) (Hirschberger et al., 2018). Premature birth is associated with reduced volume in both cerebral white and gray matter, and the period of greatest vulnerability to white matter injury is 23–32 weeks (Back, 2017). Preterm brains are particularly susceptible to cerebral white matter damage, characterized by diffuse white matter damage with aberrant regeneration of oligodendrocytes and disturbances for myelination. Among individuals born preterm, white matter damage is strongly associated with neurodevelopmental impairment, but specific treatment methods are not available for this disease. Among individuals born extremely preterm, neonatal cerebral white matter damage has been associated with an increased risk of autism spectrum disorder (Dean et al., 2016), cognitive impairment, epilepsy, and cerebral palsy (Campbell et al., 2021).

Increasing evidence suggests that the placenta plays a critical role in programming early life origins of disease, including neurodevelopmental impairment (Meakin et al., 2018; Tilley et al., 2018; Santos et al., 2020). During fetal development, the placenta serves as a critical transient organ and a conduit between the mother, the environment, and the fetus. Supporting the connection between the placenta and brain, placental inflammation has been associated with white matter damage in preterm neonates (Korzeniewski et al., 2014). Inflammatory signals derived from the placenta including cytokines, chemokines, can influence the developing neurons of the fetal brain (Yockey and Iwasaki, 2018). Gene expression in the placenta has been associated with intellectual and social impairment predictability (Santos et al., 2020), and genome-wide CpG methylation has been related to cognitive impairment (Tilley et al., 2018). In addition, epigenetic processes such as DNA methylation and genome-wide gene and miRNA expression in the placenta have been related to infant neurodevelopment (Lester and Marsit, 2018). Due to the complexity of capturing robust RNA information from the placenta, few studies have investigated the relationship between mRNA abundance and neurodevelopment. One potential molecular mechanism that could underlie the association between altered expression of placental mRNA transcripts and white matter damage in the neonate is systemic and neuroinflammation in the fetus that disrupts oligodendrocytes development and impairs myelination specifically in the preterm brain (Back and Miller, 2014; Van Steenwinckel et al., 2014).

In the present study we set out to examine the relationship between genome-wide transcript levels in the placenta, and cerebral white matter damage in the Extremely Low for Gestational Age Newborn (ELGAN) cohort. We hypothesized that transcripts that encode for proteins that are involved in proinflammatory processes would be highly expressed in the placentas of ELGANs who developed ultrasound-defined indicators of white matter damage. This research supports and contributes to increasing the understanding of the “developmental origins of health and disease” hypothesis by examining *in utero* transcriptomic changes in association with neonatal white matter damage.

## Materials and methods

### Extremely low for gestational age newborn study cohort

The ELGAN study enrolled participants from 2002 to 2004 at 14 participating medical centers across the U.S. and was approved by the Institutional Review Board at each participating center. The eligibility criterion for the ELGAN study was gestational age less than 28 weeks. Study procedures have been comprehensively described (O’Shea et al., 2009). The current analysis is based on a subsample of 279 children with data on at least two neonatal cranial ultrasounds, demographic data, and placental mRNA data.

### Placental mRNA expression data

The methods for placental DNA and RNA extraction have been detailed in prior publications (Addo et al., 2019; Santos et al., 2019). The methodology for the RNA sequencing is detailed in Eaves et al. (2020). Briefly, the AllPrep DNA/RNA/miRNA Universal kit was used to extract (Qiagen- Venlo, The Netherlands) RNA molecules ≥18 nucleotides and RNA quality was assessed using a LabChip (Perkin Elmer, Waltham, MA, USA) instrument and RNA integrity numbers (RIN) determined. The isolated placental RNA samples from ELGANs were used to measure genome-wide mRNA expression profiles using the QuantSeq 3′ mRNA-Seq Library Prep Kit (Illumina, San Diego, CA). Libraries were pooled and sequenced (single-end 50 bp) on one lane of the Illumina Hiseq 2500 and the count of sequencing reads per mRNA were aligned to the GENCODE database v3 (Hodyl et al., 2011) and organized using Salmon (Stark et al., 2011). This process yields 37,268 unique human RNA transcripts, including protein-coding and non-coding RNAs. The resulting summarized count data were then used in data processing and statistical analyses. Additional quality control steps were included to optimize the final results including: (1) filtering out non-detectable transcripts (detailed below); (2) incorporating surrogate variables in the statistical modeling approach to account for other sources of heterogeneity (detailed below); and (3) confirming previously published placental mRNA transcripts were present in the data. Specifically, Eaves et al. (2020) compared the mRNA data from the present study with the most abundant mRNAs from an independent placental whole genome RNA-sequencing study (Saben et al., 2014), resulting in a 96% overlap between the mRNA transcripts and the mRNA transcripts. Details of this analysis can be found in Eaves et al. (2020).

### Maternal and newborn characteristics

Maternal characteristics were previously ascertained *via* interview of the mother within a few days of delivery. Data collected during this interview included maternal race, education, and eligibility for public assistance, as well as the mother’s height and weight prior to the ELGAN pregnancy. Maternal medical records were reviewed by trained research assistants to collect information about pre-natal maternal health, pregnancy complications, medications taken during pregnancy, and medical treatments provided to mothers. The gestational age estimates were based on a hierarchy of the quality of available information. Prioritized were estimates based on the dates of embryo retrieval or intrauterine insemination or fetal ultrasound before the 14th week (62%). When these were not available, reliance was placed sequentially on a fetal ultrasound at 14 or more weeks (29%), last menstrual period without fetal ultrasound (7%), and gestational age recorded in the log of the neonatal intensive care unit (1%) (Addo et al., 2019).

### Cranial ultrasounds

During study participants’ initial hospitalizations (typically, the first 3–4 postnatal months), ultrasounds were collected as a component of routine clinical care. Extensive efforts, described elsewhere, were directed toward enhancing the reliability of radiologists’ interpretations of ultrasound findings interpretations of cerebral white matter injury, as indicated by either ventriculomegaly or echolucency (O’Shea et al., 1993; Kuban et al., 2007), which are the outcomes in this study. A minimum of two sonologists read the scans and for any scans that were not 100% concordant, a third sonologist resolved the discrepancy (Addo et al., 2019; Eaves et al., 2020).

### Data processing and analytical methods

To identify the placental mRNAs associated with cerebral white matter damage, mRNA sequencing data was processed using R (v 3.6.2)^1^. Similarly, to our previously published genome-wide mRNA analyses (Eaves et al., 2020), mRNA count data were normalized by median signal intensity using algorithms within DESeq2 (v1.24.0) to produce variance-stabilized expression counts (Love et al., 2014). Differential mRNA expression analysis was performed with an exclusion criterion that account for: (1) low expression values; (2) sample outliers identified through principal component analysis (PCA); and (3) missing demographic information. Prior to differential gene expression testing, batch effect and cell type differences were accounted for using surrogate variable analysis (SVA) within the SVA R package (v3.32.1) (Leek and Storey, 2007). This resulted in a total of *n* = 11,981 mRNA analysis-ready transcripts. In these models, the dependent variables were white matter damage measures of ventriculomegaly or echolucency and the independent variables were each of the 11,981 mRNA expression levels.

Known and potential hidden confounders for gene expression were estimated using the SVA approach (Leek and Storey, 2007) to identify significant (FDR-corrected *p* < 0.1) associations between mRNA transcripts and white matter damage variables. Models were also adjusted for potential confounders defined as variables that are associated with both placental gene expression and white matter damage. These included: maternal age (years; continuous), maternal education (12, 13–15, ≤16 years; categorical), insurance status (Medicaid/no Medicaid; binary), fetal sex (male/female; binary), newborn gestational age at delivery (days; continuous), and newborn birth weight z scores (<−2, <−1, ≥−1; categorical). In the primary model (Model 1), race was excluded from the analysis, as self-reported race does not represent a biological variable. Its inclusion has been met with controversy (Ioannidis et al., 2021). Nevertheless, to address this potential confounder, a secondary analysis (Model 2) included the aforementioned variables as well as race (White, Black, Other; categorical) and ethnicity (Hispanic/non-Hispanic; binary).

### Pathway and protein-protein network enrichment analyses

We examined the established functional relationships among the proteins encoded by the identified genes and their biological pathways using the Ingenuity Pathway Analysis (IPA, Ingenuity Systems^®^, Redwood City, CA, USA) and STRING v10.0 (QIAGEN IPA, n.d.; Szklarczyk et al., 2015). Enriched canonical pathways were defined as those containing more cerebral white matter damage-associated mRNAs than expected by random chance, based on a BH-corrected *p*-value calculated from a right-tailed Fisher’s Exact Text. The IPA relationships were only considered if they had been experimentally observed. Pathways with enrichment BH-corrected *p*-values < 0.05 were considered significant (Clark et al., 2021). To understand the mechanisms of transcriptional regulation underlying the observed changes in gene expression of mRNAs, the upstream regulator analysis (URA) in IPA to identify transcriptional regulators of those genes. URA uses information about the direction of the gene expression to provide an activation z-score to measure the likelihood of certain molecules to serve as regulators based on statistically significant matched patterns of down- and up-regulation using IPA libraries. The URA is also able to predict activation state of a putative regulator, which can be either activated or inhibited. Analysis was restricted to only include genes, RNAs, and proteins. We analyzed and reported canonical pathways, diseases and disorders, and molecular and cellular functions from IPA and protein domains from STRING enriched among these gene sets. To capture ASD-implicated proteins in our study, protein lists were cross-referenced with the Simons Foundation Autism Research Initiative (SFARI) gene database (Abrahams et al., 2013).

## Results

### Summary of study participants

Among surviving ELGAN participants (*n* = 1506), 379 (25.2%) had the required demographic data, placental mRNA data, and ultrasound data. General characteristics of the study participants from the full ELGAN cohort and the study sub-cohort are summarized in Table 1. Among the sub-cohort analyzed in the present study, a total of 110 (29%) self-identified as Black and 232 (61.2%) as White. Two hundred and one (53%) were male and 178 (47%) were female. The majority of infants were born between 25 and 26 weeks. Overall, 54 (14.2%) had white matter damage defined as either ventriculomegaly, echolucency or both as determined *via* ultrasound assessed during the neonatal period (birth-2 months of life). Demographic characteristics were largely similar between the full ELGAN cohort and the study sub-cohort for race and ethnicity, sex and gestational age.

**TABLE 1:** Maternal demographics, pregnancy characteristics, and neonatal outcomes among study participants.

Maternal and pregnancy characteristics		[Full ELGAN cohort]	[Study sub-cohort]
		
*N* (%)		*n* = 1,506	*n* = 379
Racial identity (self-reported)	Black	427 (28.4)	110 (29.0)
	White	866 (57.5)	232 (61.2)
	Other	187 (12.4)	35 (9.23)
	Missing	26 (1.73)	2 (0.53)
Hispanic ethnicity (self-reported)	Yes	179 (11.9)	32 (8.44)
	No	1,313(87.2)	347 (91.6)
	Missing	14 (0.93)	0(0%)
Sex	Female	707 (46.9)	178 (47.0)
	Male	799 (53.1)	201 (53.0)
Gestational age completed weeks (days)	23–24 (161–168)	409 (27.2)	88 (23.2)
	25–26 (175–182)	661 (43.9)	163 (43.0)
	27 (189)	436 (29.0)	128 (33.8)
White matter disease (ventriculomegaly and/or echolucency)	Yes	230 (15.3)	54 (14.2)
	No	1,225(81.3)	325 (85.8)
	Missing	51 (3.39)	0 (0)
Ventriculomegaly	Yes	172 (11.4)	45 (11.9)
	No	1,283(85.2)	334 (88.1)
	Missing	51 (3.39)	0 (0)
Echolucency	Yes	113 (7.50)	25 (6.60)
	No	1,342(89.1)	354 (93.4)
	Missing	51 (3.39)	0 (0)

Data are presented as the number (%) of subjects in this sub-cohort (*n* = 379), relative to the full ELGAN cohort (*n* = 1,506). Distributions of select characteristics among study participants of the Extremely Low Gestational Age Newborns Cohort, 2002–2004. Maternal demographic data, pregnancy characteristics, and data on birth outcomes are presented for the ELGAN subjects used in each analysis. Data are presented as the number (%) of subjects in the cohort. Note that some subjects have both ventriculomegaly and echolucency, so the number of subjects with ventriculomegaly and or echolucency is not intended to be equivalent to the summation of ventriculomegaly and echolucency.

### Placental mRNA transcripts among extremely low gestational age newborns are associated with white matter damage

**FIGURE 1:**
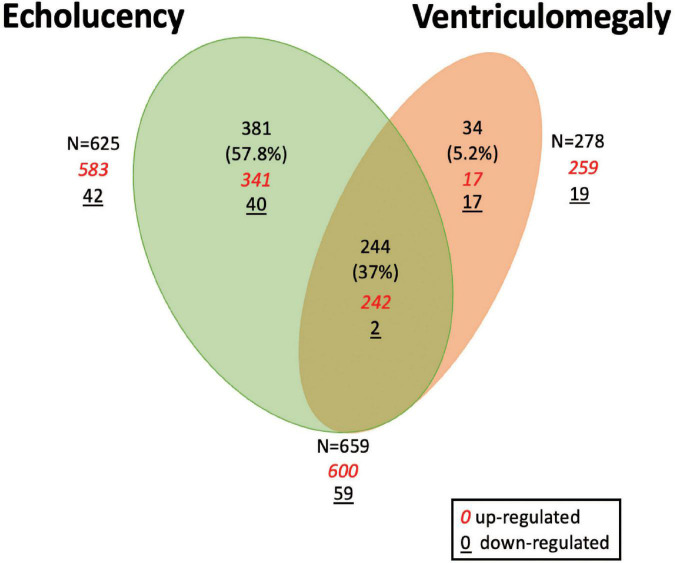
Venn diagram of differentially expressed genes in the placenta in relation to cerebral white matter damage. Venn diagram demonstrating differentially expressed placenta genes in relation to echolucency, ventriculomegaly or both. A total of 625 genes displayed associations with echolucency, 278 genes displayed associations with ventriculomegaly, and 244 genes were displayed associations with both echolucency and ventriculomegaly.

**FIGURE 2:**
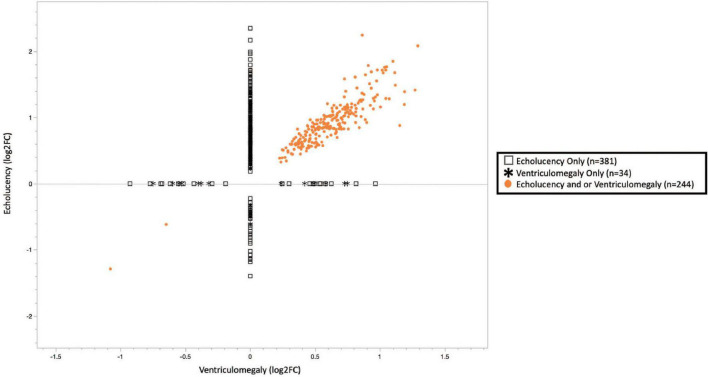
Plot of cerebral white matter damage-associated genes (*n* = 659). Similar patterns of gene expression were identified for the common gene set (*n* = 244), 242 or 99.2% genes had increased expression while 2 or 0.8% had decreased expression. While 63% of the echolucency and ventriculomegaly-associated genes displayed distinct expression levels. Log2-fold changes in mRNA expression associated with ventriculomegaly vs. echolucency. Data points colored in orange indicate genes significantly upregulated or downregulated (FDR *p*-value < 0.1) associated with both ventriculomegaly and or echolucency, data points denoted by an open square indicate genes significantly (FDR *p*-value < 0.1) associated with echolucency, and data points denoted by a star indicate genes significantly (FDR *p*-value < 0.1) associated with ventriculomegaly.

A total of 659 genes were identified in the placenta that showed an association of their expression levels with either echolucency or ventriculomegaly. A common set of 244 of the 659 genes or 37% were associated with both echolucency and ventriculomegaly (Figure 1). Of these 244 common genes, 242 (99%) showed increased expression and two showed decreased expression (Figure 2 and Supplementary Table 1a).

Echolucency was associated with the altered expression of 625 genes in placental tissue (Figure 1 and Supplementary Table 1a). Of these genes, the majority (*n* = 583) displayed increased expression in relation to echolucency, while 42 genes displayed decreased expression (Figure 1 and Supplementary Table 1a). Of these 625 echolucency-associated genes, 381 were unique (e.g., not identified in relation to ventriculomegaly) to this form of white matter damage. For these 381 echolucency unique genes, the majority (*n* = 341 or 89.5%) displayed increased expression and 40 displayed decreased expression (Figure 2 and Supplementary Table 1a).

Ventriculomegaly was associated with the altered expression of a total of 278 genes in placental tissue (Figure 1 and Supplementary Table 1a). Of these, the majority (*n* = 259 or 93%) displayed increased expression, while 19 genes displayed decreased expression (Figure 1 and Supplementary Table 1a). Of the 278 ventriculomegaly-associated genes, 34 were unique (e.g., not identified in relation to echolucency) to this form of white matter damage. For these 34 ventriculomegaly unique genes, 17 displayed increased expression and 17 displayed decreased expression (Figure 2 and Supplementary Table 1a).

In an alternate approach, statistical models that included self-reported race and ethnicity were run (Model 2). Similar to Model 1, this resulted in a total of 659 placental genes with altered expression. In relation to both indicators of white matter damage, a total of 231 (35%) genes were associated with altered expression (Supplementary Table 1a). There was a 93% (*n* = 226) overlap between Model 1 (*n* = 244) and Model 2 (*n* = 231) detailed in Supplementary Table 1a.

### Biological pathway, canonical pathway, and molecular and cellular function enrichment analysis reveal placental signatures of white matter damage

Analysis of the common set of 244 genes resulted in the identification of the enrichment of numerous key canonical pathways (*p*-values ≤ 0.05). The top five included: eukaryotic translation Initiation Factor 2 (eIF2) Signaling, Mammalian target of rapamycin (*mTOR*) Signaling, Regulation of eIF4 and p70S6K Signaling, DNA Double-Strand Break Repair by Homologous Recombination, and Interleukin 6 (Il-6) (Table 2 and Supplementary Table 1b). Interleukin 1 receptor 1 (*IL1R1*), which displayed increased expression in relation to both forms of white matter damage, is one of the genes identified as a part of the IL-6 pathway (Supplementary Table 1b). Specifically, *IL1R1* displayed a log2 fold change (FC) of +0.56 in relation to echolucency and a log2 FC of +0.41 in relation to ventriculomegaly. Diseases and disorders that were enriched among the common set of white matter damage-associated genes, include those related to organismal injury and abnormalities, cancer, endocrine system disorders, neurological disease, inflammatory response, and inflammatory and immunological disease (*p*-values ≤ 0.01) (Supplementary Table 2a). A notable gene identified from the diseases and disorders analysis included heat shock protein A5 (*HSPA5*), which is expressed in response to a range of cellular stressors (Supplementary Table 2a). Specifically, *HSPA5* displayed a log2 fold change (FC) of +0.65 in relation to echolucency and a log2 FC of +0.42 in relation to ventriculomegaly (Supplementary Table 2a). Molecular and cellular functions that were enriched among common set of the white matter damage-associated genes included RNA damage and repair, cell death and survival among others (Supplementary Table 2b).

**TABLE 2:** Top canonical pathways enriched among the 244 common genes.

Canonical pathways	*P*-value	Downregulated	Upregulated	Encoded proteins
EIF2 signaling	5.40E-13	0/212 (0%)	20/212(8%)	EIF3I, FAU, HSPA5, PIK3C2A, PPP1R15A, PTBP1, RALA*, RALB, RAP1B, RPL12, RPL13A, RPL24, RPLP2, RPS10, RPS11, RPS8, RPSA
mTOR signaling	4.37E-06	0/204 (0%)	12/204(6%)	DGKZ, EIF3I, FAU, PIK3C2A, RALA*, RALB, RAP1B, RHOQ, RPS10, RPS11, RPS8, RPSA
Regulation of eIF4 and p70S6K signaling	5.85E-06	0/175 (0%)	11/175(6%)	EIF3I, FAU, ITGA1, PIK3C2A, RALA*, RALB, RAP1B, RPS10, RPS11, RPS8, RPSA
DNA double-strand break repair by homologous recombination	3.48E-07	0/14 (0%)	5/14(29%)	ATRX, BRCA2*, MRE11, RAD50
Hereditary breast cancer signaling	4.73E-06	0/139 (0%)	9/139(6%)	ARID1A, BRCA2*, MRE11, PIK3C2A, RAD50, RALA*, RALB, RAP1B, XPC*
IL-6 signaling	1.16E-04	0/128 (0%)	8/128(6%)	IL1R1, MAPKAPK2, MCL1, PIK3C2A, PTPN11, RALA*, RALB, RAP1B

Autism spectrum disorder (ASD) genes within the study are denoted by an *.

The 381 echolucency unique genes were enriched for numerous canonical pathways including the follow top five: Vitamin D receptor (VDR) and retinoid X receptor (RXR) Activation, Glycoprotein VI Platelet (*GP6*) Signaling Pathway, Role of octamer-binding transcription factor 4 (*OCT4*) in Mammalian Embryonic Stem Cell Pluripotency, Retinoic Acid Receptor RAR Activation, and Growth Hormone Signaling (Table 3 and Supplementary Table 1c). For these canonical pathways, the following gene expression patterns were observed: within the Growth Hormone Signaling Pathway, a decrease in insulin like growth factor 2 (*IGF2*). Specifically, *IGF2* displayed a log2 fold change (FC) of −0.77 in relation to echolucency. Also, from the Growth Hormone Signaling Pathway was insulin like growth factor binding protein 3 (*IGFBP3*) which displayed a log2 fold change (FC) of +0.83 in relation to echolucency (Supplementary Table 1c). A total of 25 diseases and disorders were enriched among the echolucency unique genes including the following: organismal injury and abnormalities, cancer, endocrine system disorders, neurological disease, and inflammatory and immunological disease (*p*-values ≤ 0.05) (Supplementary Table 3a). There were 21 molecular and cellular functions that were enriched within the distinct set of echolucency-associated genes including cellular growth and proliferation, cell death and survival among others (Supplementary Table 3b).

**TABLE 3:** Top canonical pathways enriched among the 381 echolucency unique genes.

Canonical pathways	*P*-value	Downregulated	Upregulated	Encoded proteins
VDR/RXR activation	2.50E-03	1/77 (1.3%)	5/77 (6.5%)	IGFBP3, KLF4, MED1, PRKCA*, PRKCZ, SPP1
GP6 signaling pathway	6.80E-03	1/124 (0.8%)	6/124 (4.8%)	COL4A1, LAMA5, LAMB1*, LAMC1, LAMC3, PRKCA, PRKCZ
Role of OCT4 in mammalian embryonic stem cell pluripotency	8.27E-03	0/45 (0%)	4/45 (8.9%)	IGF2BP1, NR2F1, REST, SPP1
RAR activation	8.40E-03	0/195 (0%)	9/195 (4.6%)	BRD7, IGFBP3, MED1, NR2F1*, PRKCA*, PRKCZ, PRMT2, SMARCC1, SMARCE1
Growth hormone signaling	8.66E-03	1/71 (1.4%)	4/71 (5.6%)	CSH1/CSH2, IGF2, IGFBP3, PRKCA*, PRKCZ

Autism spectrum disorder (ASD) genes within the study are denoted by an *.

Pathway analysis of the 34 ventriculomegaly unique genes highlighted the following top five canonical pathways, Tumor protein P53 (p53) Signaling, MicroRNA Biogenesis Signaling Pathway, Integrin-linked kinase (ILK) Signaling, phosphatidylinositol 3-kinase (PI3K)/protein kinase B (AKT) Signaling, and Integrin Signaling (Table 4 and Supplementary Table 1d). Within the P53 Pathway, a decrease in the expression of glycogen synthase kinase 3 beta (*GSK3B*) (log2 FC: −0.43) was observed. Organismal injury and abnormalities, cancer, endocrine system disorders, neurological disease, inflammatory response, and immunological disease (*p*-values ≤ 0.05) were significantly enriched in the ventriculomegaly unique genes (Supplementary Table 3c). Among the set of unique ventriculomegaly genes, we identified 21 enriched molecular and cellular functions that included cellular function and maintenance, cell-to-cell signaling and interaction and others (Supplementary Table 3d).

**TABLE 4:** Top canonical pathways enriched in the 34 ventriculomegaly unique genes.

Canonical pathways	*P*-value	Downregulated	Upregulated	Encoded proteins
P53 signaling	4.96E-04	2/98 (2.0%)	1/98 (1%)	GSK3B, ST13, STAG1*
MicroRNA biogenesis signaling pathway	2.99E-03	1/183 (0.5%)	2/183 (1.1%)	DDX5, HSP90AB1, ST12
ILK signaling	3.63E-03	2/196 (1.0%)	1/196 (0.5%)	GSBK3B, ITGB2, RHOBTB1
PI3K/AKT signaling	3.73E-03	1/198 (0.5%)	2/198 (1%)	GSK3B, HSP90AB1, ITGB2
Integrin signaling	4.11E-03	2/205 (1%)	1/205 (0.5%)	GSBK3B, ITGB2, RHOBTB1

Autism spectrum disorder (ASD) genes within the study are denoted by an *.

### Upstream regulator analyses highlight potential regulatory mechanisms underlying white matter-associated gene expression changes

**FIGURE 3:**
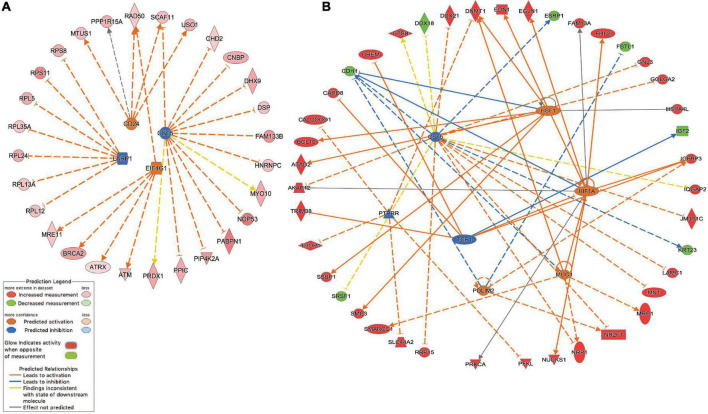
Inhibited/activated upstream regulators and their target genes. **(A)** An integrated network *among* the common set of *cerebral white matter associated genes* (*n* = 244) *that includes* La ribonucleoprotein 1 (*LARP1)*, CD24 molecule *(CD24)*, Cystatin D (*CST5)*, and eukaryotic translation initiation factor 4 gamma 1 (*EIF4G1);* **(B)** An integrated network *among the echolucency unique genes (n = 381*) that includes *CST5*, Hypoxia-inducible factor 1-alpha (*HIF1A*), mucin 1, cell surface associated (*MUC1*), PDZ and LIM Domain 2 (*PDLIM2*), *PTPRR*, and transcription factor 4 (*TCF4*) *1.* All the target genes were differentially expressed based on the meta-analysis. For example, the activation of *EIF4G1* leads to the overexpression (indicated by the orange arrow line) of alpha thalassemia/mental retardation syndrome X-linked (*ATRX*) (indicated by the red color). For other indicators, please refer to the Prediction Legend.

Network analysis identified 64 protein-protein interactions among the 244 common white matter damage-associated genes (Supplementary Figure 1). The identified network has significantly more interactions than expected by chance (protein-protein interaction enrichment *p*-value: 2.11e-11). To gain an understanding of transcriptional regulators that may underly the expression of the common gene set, IPA upstream regulator analysis was performed, identifying 131 significant upstream regulators (*p*-value < 0.05) (Supplementary Table 4a). Among those upstream regulators is eukaryotic translation initiation factor 4 gamma 1 (*EIF4G1)* and CD24 molecule *(CD24)* were identified as predictively activated regulators (*z*-score = 2.24 and 2.00, respectively). In contrast, La ribonucleoprotein 1 (*LARP1)* and cystatin D (*CST5)* were identified as predictively inhibited regulators (*z*-scores = −2.65 and −2.50, respectively). Figure 3a highlights these four upstream regulators and their target genes.

Network analysis identified 106 protein-protein interactions among the 381 echolucency unique-associated genes (Supplementary Figure 2). The identified network has significantly more interactions than expected by chance (protein-protein interaction enrichment *p*-value: 0.004). Additionally, 241 significant upstream regulators were identified from the echolucency unique gene set (Figure 3b). These included Cystatin D (*CST5)*, protein tyrosine phosphatase receptor type R (*PTTPRR*), and transcription factor 4 (*TCF4*) (*z*-scores = −2.21, −2.22, and −2.24, respectively) were predicted to be inhibited relative to echolucency, while mucin 1, cell surface associated (*MUC1*), heat shock transcription factor 1(*HFS1*), Hypoxia-inducible factor 1-alpha (*HIF1A*) and PDZ and LIM Domain 2 (*PDLIM2*) were predicted to be activated (*z*-scores = 2.19, 2.43, 2.39, and 2.0, respectively). Another notable upstream regulator identified from the echolucency gene set is estrogen receptor 1 (ESR1) (Supplementary Table 4b).

Network analysis identified two protein-protein interactions among the 34 ventriculomegaly unique genes (Supplementary Figure 2). The identified network did not have significantly more interactions than expected by chance (protein-protein interaction enrichment *p*-value: 0.55). A total of 52 significant upstream regulators were identified from the ventriculomegaly unique genes (*p*-value < 0.05). Due to the small number, predicted activation and inhibition of upstream regulator information was unavailable for the ventriculomegaly unique gene set (Supplementary Table 4c).

### Autism spectrum disorder and endocrine system disorder associated biological processes identified among the white matter damage-associated genes

To identify whether any of the white matter damage-associated genes from the present study (*n* = 659) have known alterations in neurodevelopmental disease states, we compared these genes to a list of *n* = 1075 autism spectrum disorder (ASD)-associated genes obtained from the SFARI database. Numerous (*n* = 63, 3.8%) ASD-associated genes were found including common (*n* = 24/244), echolucency unique (*n* = 31/381), and ventriculomegaly unique (*n* = 1/34) genes. These genes included laminin subunit beta 1(LAMB1), ATPase Na+/K+ transporting subunit alpha 1 (*ATP1A1*), alpha thalassemia/mental retardation syndrome X-linked (*ATRX*), and methyl-CpG binding domain protein 1 (*MBD1*) (Supplementary Table 1a).

Furthermore, to identify whether any of the white matter damage-associated genes from the present study (*n* = 659) have known alterations in the endocrine system, we analyzed these genes within the IPA database. A total of 170 endocrine system disorder genes were differentially expressed in the placenta in relation to the common gene set (*n* = 71/244) and also the echolucency unique genes (*n* = 99/381) (Supplementary Table 1a). Interestingly, these genes also included the ASD-associated genes *IGFBP3*, *ATRX*, *LAMB1* and *MBD1* (Supplementary Table 1a).

**FIGURE 4:**
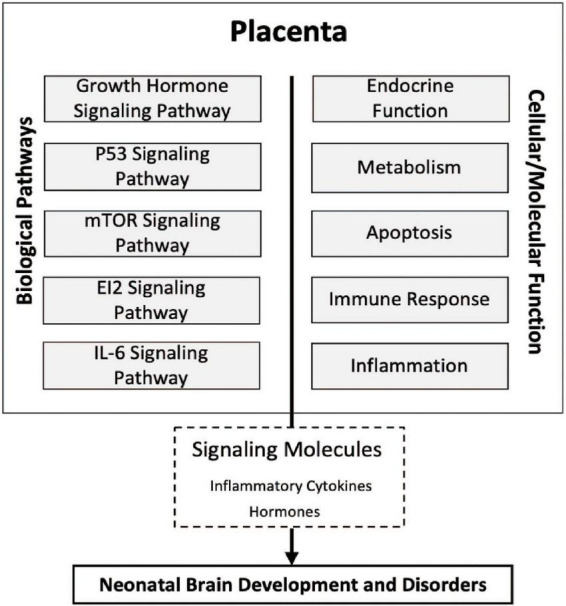
Conceptual diagram of the study findings. Relationships among molecular processes associated with cerebral white matter damage that may provide insight into the physiologic state of the placentas, transcriptomic pathways, and neonatal brain development. Five novel signaling pathways eukaryotic translation initiation factor 2 (eIF2), interleukin-6 (IL-6), and mammalian target of rapamycin (mTOR), growth hormone, and tumor protein P53 (p53) in relation to ventriculomegaly associated cerebral white matter brain damage relatively early in life. Many of the cellular/molecular processes are associated with more than one signaling pathway and there may be shared overlap of many of the genes within a given pathway.

Taken together, the results of the present study highlight the association of the expression of genes involved in the eIF2, mTOR, IL-6, growth hormone, and P53 signaling pathways within the placenta as a contributor to white matter damage. These pathways are broadly tied to apoptosis, inflammation, immune response, hormone disruption, and metabolism. The dysregulation of these cellular and molecular processes within these placental pathways *in utero* may modify the release of signaling molecules such as inflammatory cytokines and hormones that ultimately lead to neonatal brain development and disorders in children born extremely preterm intriguing (Figure 4).

## Discussion

Strong evidence supports that placental physiology is tied to neurodevelopment later in life, a concept known as the placenta-brain-axis (Behura et al., 2019). Nevertheless, linkages between the placenta, placental gene expression, and white matter damage in the neonate are understudied. To address this, we examined whether transcript levels of specific genes in the placenta are related to two forms of white matter damage in the neonate. The white matter damage was identified by two ultrasound-defined indicators of cerebral white matter damage in the ELGAN cohort. In addition to supporting our hypothesis that inflammation-related genes in the placenta would be associated with white matter damage, the transcriptomic signatures highlighted other biological processes in the placenta. These include immune response, apoptosis, metabolism, and hormone-related signaling in relation to echolucency and ventriculomegaly. These results are clinically relevant as cerebral white matter injury occurs in 12% of ELGAN survivors and is associated strongly with ASD (Dean et al., 2016), cerebral palsy (Kuban et al., 2009) and cognitive impairment (Campbell et al., 2021).

Expression of a total of 659 genes in the placenta was associated with echolucency, and/or ventriculomegaly. Of these genes, a set of 244 genes displayed expression levels that were related to both forms of white matter damage. The small overlap (37%) between echolucency and ventriculomegaly-associated transcripts was not unexpected as there may be etiologic pathways that are not shared by these two indicators of white matter damage. The transcripts that were part of the common gene set encode proteins involved in proinflammatory processes and were highly expressed in the placentas of individuals who had cerebral white matter damage. Among the differentially expressed transcripts were those involved broadly in the Interleukin 1 (IL-1) and Interleukin 6 (IL-6) pathways. These pathways which are critical for the modulation of the immune system and coordinating cell-mediated immune responses (Hirano, 2020). The identification of these pathways support our *a priori* hypothesis that inflammation-associated genes would be dysregulated in the placenta of ELGANs with white matter damage. Interestingly, inflammation in the placenta has been tied to neurodevelopment later in life (Korzeniewski et al., 2014). In addition to inflammation-related genes in the common gene set, we also found genes in the eIF2 and mTOR signaling pathways that were enriched. These pathways are broadly tied to apoptosis, and immune response. Specifically, the mTOR pathway is critical for cellular growth, metabolism and apoptosis (Saxton and Sabatini, 2017). Apoptosis in the placenta has been tied to hypoxia, oxidative stress and neurodevelopmental outcomes (Sharp et al., 2010; Marks et al., 2020). The altered expression of these pathways may point to improper placental growth and invasion which would directly impact placental development and function (Raguema et al., 2020).

By uncoupling the echolucency and ventriculomegaly-specific gene expression we observed the enrichment of apoptosis-related pathways in the placenta as they relate specifically to ventriculomegaly. Among the pathways enriched in the ventriculomegaly unique gene set was the P53 signaling pathway. This pathway which is related to mTOR and plays an intrinsic role in apoptotic and metabolic processes. In support of these data, alterations during central nervous system (CNS) development such as overexpression and inhibition of P53 pathway genes contribute to cerebellar defects (Mendrysa et al., 2011). In the present analysis we observed a decrease in *GSK3B*, which is a negative regulator of glucose mobilization essential to apoptotic pathways as well as energy metabolism, and inflammation (Kulikov et al., 2005). Alterations such as this within the P53 pathway may be present as a response to DNA damage or oxidative stress in the placenta.

Additionally, the uncoupling of the placental gene expression signatures in relation to echolucency and ventriculomegaly may help to reveal genes and pathways that are related to one form of white matter damage but not the other. For example, among the pathways enriched in the echolucency unique gene set was growth hormone signaling. This pathway involves genes including *IGF2* and *IGFBP3* which displayed decreased expression in the placentae in relation to echolucency in the present study. These alterations may be involved in changes in fetal growth (Velegrakis et al., 2017). Further support for our findings are that associations have also been found between systemic inflammation measured *via IGF* family proteins relative to white matter injury (Hansen-Pupp et al., 2013).

Interestingly, when analyzing the complete gene set of white matter damage-associated genes, genes involved in ASD and the endocrine system were identified. The latter is consistent with the known influence on neurodevelopment of several hormones produced by the placenta, such as corticotropin-releasing hormone (CRH) (Kassotaki et al., 2021), estradiol (McCarthy, 2008), and allopregnanolone (Vacher et al., 2021). Previous studies within the ELGAN cohort have highlighted the associations between differential methylation in placenta of hypothalamic-pituitary-adrenal (HPA) axis genes, such as nuclear receptor subfamily group 3CMember 1 (*NR3C1*), FK506 binding protein 5 (*FKPB5*), and brain-derived neurotrophic Factor (*BDNF*), and cognitive impairment in the offspring (Meakin et al., 2018). Dysregulation of many of these genes within the HPA axis may be associated with crucial biological functions of the placenta like nutrient transfer and cellular proliferation, and hormone production (Kawamura et al., 2009). For example, *BDNF* promotes both regulation of CRH (Gao et al., 2012; Lu et al., 2014; Meakin et al., 2018). Subsequently, characterizing the dysregulation of hormones in the placenta is an important area of investigation since prenatal hormone dysregulation may contribute to alterations in fetal neurodevelopment and ultimately ASD development.

These data are among the first to highlight the expression of these critical pathways in placentas collected from infants born extremely prematurely (<28 weeks’ gestation) who subsequently developed white matter damage. Still, this study is not without limitations. First, the ELGAN cohort includes the limited range of gestational ages in the study sample, which could limit the generalizability of the study results. Second, the present research focused on mRNA levels only in the placenta. In the future, the analysis of proteomic data could increase understanding of the relationship between molecular processes in the placenta and brain structure and function later-in-life. Fourth, neonatal cranial ultrasound identifies only macroscopic white matter damage and is less sensitive than magnetic resonance imaging for detection of white matter damage (Ciambra et al., 2013). Nevertheless, in the ELGAN study, the presence of either echolucency or ventricular enlargement was strongly associated with subsequent development of cerebral palsy (Kuban et al., 2009), epilepsy (Campbell et al., 2021), and cognitive impairment (Campbell et al., 2021) and were the ultrasound lesions most predictive of neurodevelopmental impairments. Furthermore, we understand that the scope of neurodevelopment among ELGANs and other infants who survive preterm birth cannot be accounted for solely by the presence of structural brain abnormalities. To address this, future research will incorporate functional measures of neurodevelopment data as an outcome measurement in relation to the placental epigenomic signatures identified here and other later-in-life neurodevelopmental outcomes.

In conclusion, using a unique placental repository comprising genome-wide transcript levels, we have integrated the transcript level changes of gene expression within a framework of biological pathways aimed at uncovering the physiologic state of the placenta. These data point to the gene regulation of hormones and inflammatory molecules that may have influenced the developing brain of the fetus. The results highlight biological pathways in the placenta that are associated with white matter damage among children born extremely premature. In terms of a mechanism that may underlie the observed association between the increased expression of these placental transcripts and white matter damage in the neonate, inflammatory molecules such as cytokines and hormones are known to disrupt oligodendrocytes development and impair myelination in the preterm brain (Back and Miller, 2014; Van Steenwinckel et al., 2014). In addition, the presence of placental inflammation and alterations in the associated placental transcripts may be indicators of placental toxicity and dysregulated placental development. The pathways identified in the present study may represent targets for disease intervention, through the enhanced understanding of perinatal factors that influence their expression in the placenta.

## Data availability statement

Ultrasound data are available by completing the NINDS Data Request Form (https://www.ninds.nih.gov/sites/default/files/sig_form_revised_508c.pdf), which is available from Archived Clinical Research Datasets (https://www.ninds.nih.gov/Current-Research/Research-Funded-NINDS/Clinical-Research/Archived-Clinical-Research-Datasets), and sending the completed form to CRLiaison@ninds.nih.gov. Raw and processed placental molecular sequencing data is available through the Gene Expression Omnibus (GEO) repository and are publicly available under GEO series GSE154829 (NCBI, 2020).

## Ethics statement

This research was approved by the Institutional Review Board at UNC-Chapel Hill (IRB # 16-2535). Written informed consent to participate in this study was provided by the participants’ parent or legal guardian and assent was provided by participants.

## Author contributions

CM, TO’S, and RF conceived of the presented idea. KR and CM developed the theory. CM performed the computations. KR, CM, and RF verified the analytical methods. TO’S encouraged CM to investigate neonatal ultrasound data and supervised the findings of this work. CM carried out the experiment and wrote the manuscript with support from RF and TO’S. TO’S and KK fabricated the ultrasound samples and conceived the foundation-setting idea. RF and TO’S helped to supervise the project. All authors discussed the results, contributed to the final manuscript, provided critical feedback, helped to shape the research and manuscript, and approved the submitted version.

## Footnotes

1
cran.r-project.org/

## Supplementary material

Supplementary Figure 1 Network analysis of the common gene set (*n* = 244) in the placenta. Interactome of gene–gene interactions representing joint contribution to a shared function. Protein interaction network analysis indicates the genes belonging to the eukaryotic translation initiation factor 2 (eIF2), mammalian target of rapamycin (mTOR), and interleukin-6 (IL-6) Signaling pathways playing a central role in the interactome of placenta that are associated with cerebral white matter damage. All 244 genes were used as input for STRING analysis and a network of 64 proteins was built (Supplementary Table 1a). Shown are the details of protein-protein interactions based on highest confidence (0.9) evidence from experimental protein-protein interaction (purple lines) and curated (blue lines) databases. Proteins are indicated by nodes labeled with the encoding gene symbol. Nodes of disconnected proteins were hidden. The network is enriched in interactions (*p* = 4.2E-10).

Supplementary Figure 2 Network analysis of the echolucency unique genes (*n* = 381) in the placenta. Interactome of gene–gene interactions representing joint contribution to a shared function. Protein interaction network analysis indicates the genes belonging to the growth hormone pathway playing a central role in the interactome of placenta that are associated with cerebral white matter damage. All 381 genes were used as input for STRING analysis and a network of 106 proteins was built (Supplementary Table 1a). Shown are the details of protein-protein interactions based on highest confidence (0.9) evidence from experimental protein-protein interaction (purple lines) and curated (blue lines) databases. Proteins are indicated by nodes labeled with the encoding gene symbol. Nodes of disconnected proteins were hidden. The network is enriched in interactions (*p* = 0.00405).

Supplementary Figure 3 Network analysis of the ventriculomegaly unique genes (*n* = 34) in the placenta. Interactome of gene–gene interactions representing joint contribution to a shared function. Protein interaction network analysis indicates the genes belonging to the clathrin-mediated endocytosis signaling pathway playing a central role in the interactome of placenta that are associated with cerebral white matter damage. All 381 genes were used as input for STRING analysis and a network of two proteins was built (Supplementary Table 1a). Shown are the details of protein-protein interactions based on highest confidence (0.9) evidence from experimental protein-protein interaction (purple lines) and curated (blue lines) databases. Proteins are indicated by nodes labeled with the encoding gene symbol. Nodes of disconnected proteins were hidden. The network is enriched in interactions (*p* = 0.569).

## Abbreviations

ELGAN: extremely low gestational age newborns
ASD: autism spectrum disorder
ADHD: attention deficit/hyperactivity disorder
IL: interleukin
eIF2: eukaryotic translation initiation factor 2
mTOR: mammalian target of rapamycin
IL-6: interleukin-6.

## References

[B1] Abrahams B. S. Arking D. E. Campbell D. B. Mefford H. C. Morrow E. M. Weiss L. A. (2013). SFARI Gene 2.0: A community-driven knowledgebase for the autism spectrum disorders (ASDs). *Mol. Autism* 4:36. 10.1186/2040-2392-4-36 24090431 PMC3851189

[B2] Addo K. A. Bulka C. Dhingra R. Santos H. P. Jr. Smeester L. O’Shea T. M. (2019). Acetaminophen use during pregnancy and DNA methylation in the placenta of the extremely low gestational age newborn (ELGAN) cohort. *Environ. Epigenet.* 5:dvz010. 10.1093/eep/dvz010 PMC6682751 31404209

[B3] Back S. A. (2017). White matter injury in the preterm infant: Pathology and mechanisms. *Acta Neuropathol.* 134 331–349. 28534077 10.1007/s00401-017-1718-6 PMC5973818

[B4] Back S. A. Miller S. P. (2014). Brain injury in premature neonates: A primary cerebral dysmaturation disorder? *Ann. Neurol.* 75 469–486. 24615937 10.1002/ana.24132 PMC5989572

[B5] Behura S. K. Dhakal P. Kelleher A. M. Balboula A. Patterson A. Spencer T. E. (2019). The brain-placental axis: Therapeutic and pharmacological relevancy to pregnancy. *Pharmacol. Res.* 149:104468. 10.1016/j.phrs.2019.104468 31600597 PMC6944055

[B6] Bhutta A. T. Cleves M. A. Casey P. H. Cradock M. M. Anand K. J. (2002). Cognitive and behavioral outcomes of school-aged children who were born preterm: A meta-analysis. *JAMA* 288 728–737. 10.1001/jama.288.6.728 12169077

[B7] Campbell H. Check J. Kuban K. C. K. Leviton A. Joseph R. M. Frazier J. A. (2021). Neonatal cranial ultrasound findings among infants born extremely preterm: Associations with neurodevelopmental outcomes at 10 years of age. *J. Pediatr.* 237 197–205.e4. 10.1016/j.jpeds.2021.05.059 34090894 PMC8478718

[B8] Ciambra G. Arachi S. Protano C. Cellitti R. Caoci S. Di Biasi C. (2013). Accuracy of transcranial ultrasound in the detection of mild white matter lesions in newborns. *Neuroradiol. J.* 26 284–289. 10.1177/197140091302600305 23859283 PMC5278841

[B9] Clark J. Eaves L. A. Gaona A. R. Santos H. P. Smeester L. Bangma J. T. (2021). Pre-pregnancy BMI-associated miRNA and mRNA expression signatures in the placenta highlight a sexually-dimorphic response to maternal underweight status. *Sci. Rep.* 11:15743. 10.1038/s41598-021-95051-1 34344912 PMC8333418

[B10] Dean D. C. III Travers B. G. Adluru N. Tromp do P. M. Destiche D. J. Samsin D. (2016). Investigating the microstructural correlation of white matter in autism spectrum disorder. *Brain Connect* 6 415–433. 27021440 10.1089/brain.2015.0385 PMC4913512

[B11] Dimes M. O. (2021). *Fighting premature birth: The prematurity campain.* Available online at: https://www.marchofdimes.org/mission/prematurity-campaign.aspx (accessed August 26, 2021).

[B12] Eaves L. A. Phookphan P. Rager J. E. Bangma J. Santos H. P. Jr. Smeester L. (2020). A role for microRNAs in the epigenetic control of sexually dimorphic gene expression in the human placenta. *Epigenomics* 12 1543–1558. 10.2217/epi-2020-0062 32901510 PMC7607407

[B13] Gao L. Lv C. Xu C. Li Y. Cui X. Gu H. (2012). Differential regulation of glucose transporters mediated by CRH receptor type 1 and type 2 in human placental trophoblasts. *Endocrinology* 153 1464–1471. 10.1210/en.2011-1673 22234467

[B14] Hansen-Pupp I. Hövel H. Löfqvist C. Hellström-Westas L. Fellman V. Hüppi P. S. (2013). Circulatory insulin-like growth factor-I and brain volumes in relation to neurodevelopmental outcome in very preterm infants. *Pediatr. Res.* 74 564–569. 10.1038/pr.2013.135 23942554

[B15] Hirano T. (2020). IL-6 in inflammation, autoimmunity and cancer. *Int. Immunol.* 33 127–148. 10.1093/intimm/dxaa078 PMC7799025 33337480

[B16] Hirschberger R. G. Kuban K. C. K. O’Shea T. M. Joseph R. M. Heeren T. Douglass L. M. (2018). Co-occurrence and Severity of neurodevelopmental Burden (Cognitive Impairment, Cerebral Palsy, Autism Spectrum Disorder, and Epilepsy) at age ten years in children born extremely preterm. *Pediatr. Neurol.* 79 45–52. 10.1016/j.pediatrneurol.2017.11.002 29310907 PMC5803305

[B17] Hodyl N. A. Stark M. J. Osei-Kumah A. Clifton V. L. (2011). Prenatal programming of the innate immune response following in utero exposure to inflammation: A sexually dimorphic process? *Expert Rev. Clin. Immunol.* 7 579–592. 10.1586/eci.11.51 21895471

[B18] Ioannidis J. P. A. Powe N. R. Yancy C. (2021). Recalibrating the use of race in medical research. *JAMA* 325 623–624. 33492329 10.1001/jama.2021.0003

[B19] QIAGEN IPA (n.d.). *Data were analyzed with the use of QIAGEN IPA*. QIAGEN Inc. Available online at: https://digitalinsights.qiagen.com/IPA

[B20] Kassotaki I. Valsamakis G. Mastorakos G. Grammatopoulos D. K. (2021). Placental CRH as a signal of pregnancy adversity and impact on fetal neurodevelopment. *Front. Endocrinol.* 12:714214. 10.3389/fendo.2021.714214 34408727 PMC8366286

[B21] Kawamura K. Kawamura N. Sato W. Fukuda J. Kumagai J. Tanaka T. (2009). Brain-derived neurotrophic factor promotes implantation and subsequent placental development by stimulating trophoblast cell growth and survival. *Endocrinology* 150 3774–3782. 10.1210/en.2009-0213 19372195

[B22] Korzeniewski S. J. Romero R. Cortez J. Pappas A. Schwartz A. G. Kim C. J. (2014). A “multi-hit” model of neonatal white matter injury: Cumulative contributions of chronic placental inflammation, acute fetal inflammation and postnatal inflammatory events. *J. Perinat. Med.* 42 731–743. 10.1515/jpm-2014-0250 25205706 PMC5987202

[B23] Krämer A. Green J. Pollard J. Jr. Tugendreich S. (2014). Causal analysis approaches in ingenuity pathway analysis. *Bioinformatics* 30, 523–530. 24336805 10.1093/bioinformatics/btt703 PMC3928520

[B24] Kuban K. C. Allred E. N. O’Shea T. M. Paneth N. Pagano M. Dammann O. (2009). Cranial ultrasound lesions in the NICU predict cerebral palsy at age 2 years in children born at extremely low gestational age. *J. Child Neurol.* 24 63–72. 10.1177/0883073808321048 19168819 PMC2814246

[B25] Kuban K. Adler I. Allred E. N. Batton D. Bezinque S. Betz B. W. (2007). Observer variability assessing US scans of the preterm brain: The ELGAN study. *Pediatr. Radiol.* 37 1201–1208. 10.1007/s00247-007-0605-z 17901950 PMC2803345

[B26] Kulikov R. Boehme K. A. Blattner C. (2005). Glycogen synthase kinase 3-dependent phosphorylation of Mdm2 regulates p53 abundance. *Mol. Cell. Biol.* 25 7170–7180. 10.1128/MCB.25.16.7170-7180.2005 16055726 PMC1190268

[B27] Leek J. T. Storey J. D. (2007). Capturing heterogeneity in gene expression studies by surrogate variable analysis. *PLoS Genet.* 3:1724–1735. 10.1371/journal.pgen.0030161 17907809 PMC1994707

[B28] Lester B. M. Marsit C. J. (2018). Epigenetic mechanisms in the placenta related to infant neurodevelopment. *Epigenomics* 10 321–333. 29381081 10.2217/epi-2016-0171 PMC6219448

[B29] Love M. I. Huber W. Anders S. (2014). Moderated estimation of fold change and dispersion for RNA-seq data with DESeq2. *Genome Biol.* 15:550. 10.1186/s13059-014-0550-8 25516281 PMC4302049

[B30] Lu B. Nagappan G. Lu Y. (2014). BDNF and synaptic plasticity, cognitive function, and dysfunction. *Handb. Exp. Pharmacol.* 220 223–250. 24668475 10.1007/978-3-642-45106-5_9

[B31] Marks K. Vincent A. Coutinho E. (2020). Maternal-autoantibody-related (MAR) autism: Identifying neuronal antigens and approaching prospects for intervention. *J. Clin. Med.* 9:2564. 10.3390/jcm9082564 32784803 PMC7465310

[B32] McCarthy M. M. (2008). Estradiol and the developing brain. *Physiol. Rev.* 88 91–124. 18195084 10.1152/physrev.00010.2007 PMC2754262

[B33] Meakin C. J. Martin E. M. Santos H. P. Jr. Mokrova I. Kuban K. O’Shea T. M. (2018). Placental CpG methylation of HPA-axis genes is associated with cognitive impairment at age 10 among children born extremely preterm. *Horm. Behav.* 101 29–35. 10.1016/j.yhbeh.2018.02.007 29477804 PMC6354776

[B34] Mendrysa S. M. Ghassemifar S. Malek R. (2011). p53 in the CNS: Perspectives on development, stem cells, and cancer. *Genes Cancer* 2 431–442. 10.1177/1947601911409736 21779511 PMC3135640

[B35] NCBI (2020). *Geo gene expression omnibus. Placental genomic and epigenomic signatures in infants borns at extremely lowgestational age.* Bethesda, MD: NCBI.

[B36] O’Shea T. M. Allred E. N. Dammann O. Hirtz D. Kuban K. C. K. Paneth N. (2009). The ELGAN study of the brain and related disorders in extremely low gestational age newborns. *Early Hum. Dev.* 85 719–725. 19765918 10.1016/j.earlhumdev.2009.08.060 PMC2801579

[B37] O’Shea T. M. Volberg F. Dillard R. G. (1993). Reliability of interpretation of cranial ultrasound examinations of very low-birthweight neonates. *Dev. Med. Child Neurol.* 35 97–101. 8444332 10.1111/j.1469-8749.1993.tb11611.x

[B38] Purisch S. E. Gyamfi-Bannerman C. (2017). Epidemiology of preterm birth. *Semin. Perinatol.* 41 387–391. 28865982 10.1053/j.semperi.2017.07.009

[B39] Raguema N. Moustadraf S. Bertagnolli M. (2020). Immune and apoptosis mechanisms regulating placental development and vascularization in preeclampsia. *Front. Physiol.* 11:98. 10.3389/fphys.2020.00098 32116801 PMC7026478

[B40] Saben J. Zhong Y. McKelvey S. Dajani N. K. Andres A. Badger T. M. (2014). A comprehensive analysis of the human placenta transcriptome. *Placenta* 35 125–131. 24333048 10.1016/j.placenta.2013.11.007 PMC3978120

[B41] Santos H. P. Jr. Bhattacharya A. Joseph R. M. Smeester L. Kuban K. C. K. Marsit C. J. (2020). Evidence for the placenta-brain axis: Multi-omic kernel aggregation predicts intellectual and social impairment in children born extremely preterm. *Mol. Autism* 11:97. 10.1186/s13229-020-00402-w 33308293 PMC7730750

[B42] Santos H. P. Jr. Bhattacharya A. Martin E. M. Addo K. Psioda M. Smeester L. (2019). Epigenome-wide DNA methylation in placentas from preterm infants: Association with maternal socioeconomic status. *Epigenetics* 14 751–765. 10.1080/15592294.2019.1614743 31062658 PMC6615526

[B43] Saxton R. A. Sabatini D. M. (2017). mTOR signaling in growth, metabolism, and disease. *Cell* 168 960–976. 28283069 10.1016/j.cell.2017.02.004 PMC5394987

[B44] Sharp A. N. Heazell A. E. P. Crocker I. P. Mor G. (2010). Placental apoptosis in health and disease. *Am. J. Reprod. Immunol.* 64 159–169. 20367628 10.1111/j.1600-0897.2010.00837.x PMC3025811

[B45] Stark M. J. Hodyl N. A. Wright I. M. Clifton V. L. (2011). Influence of sex and glucocorticoid exposure on preterm placental pro-oxidant-antioxidant balance. *Placenta* 32 865–870. 10.1016/j.placenta.2011.08.010 21903264

[B46] Szklarczyk D. Franceschini A. Wyder S. Forslund K. Heller D. Huerta-Cepas J. (2015). STRING v10: Protein-protein interaction networks, integrated over the tree of life. *Nucleic Acids Res.* 43 D447–D452. 10.1093/nar/gku1003 25352553 PMC4383874

[B47] Tilley S. K. Martin E. M. Smeester L. Joseph R. M. Kuban K. C. K. Heeren T. C. (2018). Placental CpG methylation of infants born extremely preterm predicts cognitive impairment later in life. *PLoS One* 13:e0193271. 10.1371/journal.pone.0193271 29513726 PMC5841757

[B48] Vacher C.-M. Lacaille H. O’Reilly J. J. Salzbank J. Bakalar D. Sebaoui S. (2021). Placental endocrine function shapes cerebellar development and social behavior. *Nat. Neurosci.* 24 1392–1401. 10.1038/s41593-021-00896-4 34400844 PMC8481124

[B49] Van Steenwinckel J. Schang A. L. Sigaut S. Chhor V. Degos V. Hagberg H. (2014). Brain damage of the preterm infant: New insights into the role of inflammation. *Biochem. Soc. Trans.* 42 557–563. 10.1042/BST20130284 24646278

[B50] Velegrakis A. Sfakiotaki M. Sifakis S. (2017). Human placental growth hormone in normal and abnormal fetal growth. *Biomed. Rep.* 7 115–122. 28804622 10.3892/br.2017.930 PMC5526045

[B51] Wang C. Geng H. Liu W. Zhang G. (2017). Prenatal, perinatal, and postnatal factors associated with autism: A meta-analysis. *Medicine* 96:e6696. 10.1097/MD.0000000000006696 28471964 PMC5419910

[B52] Yockey L. J. Iwasaki A. (2018). Interferons and proinflammatory cytokines in pregnancy and fetal development. *Immunity* 49 397–412. 30231982 10.1016/j.immuni.2018.07.017 PMC6152841

