# Supplementary figures and images for: Placental transcriptional signatures associated with cerebral white matter damage in the neonate
Source: Front Neurosci. 2022 Oct 28;16:1017953. doi: 10.3389/fnins.2022.1017953 (PMC9650394; doi:10.3389/fnins.2022.1017953)

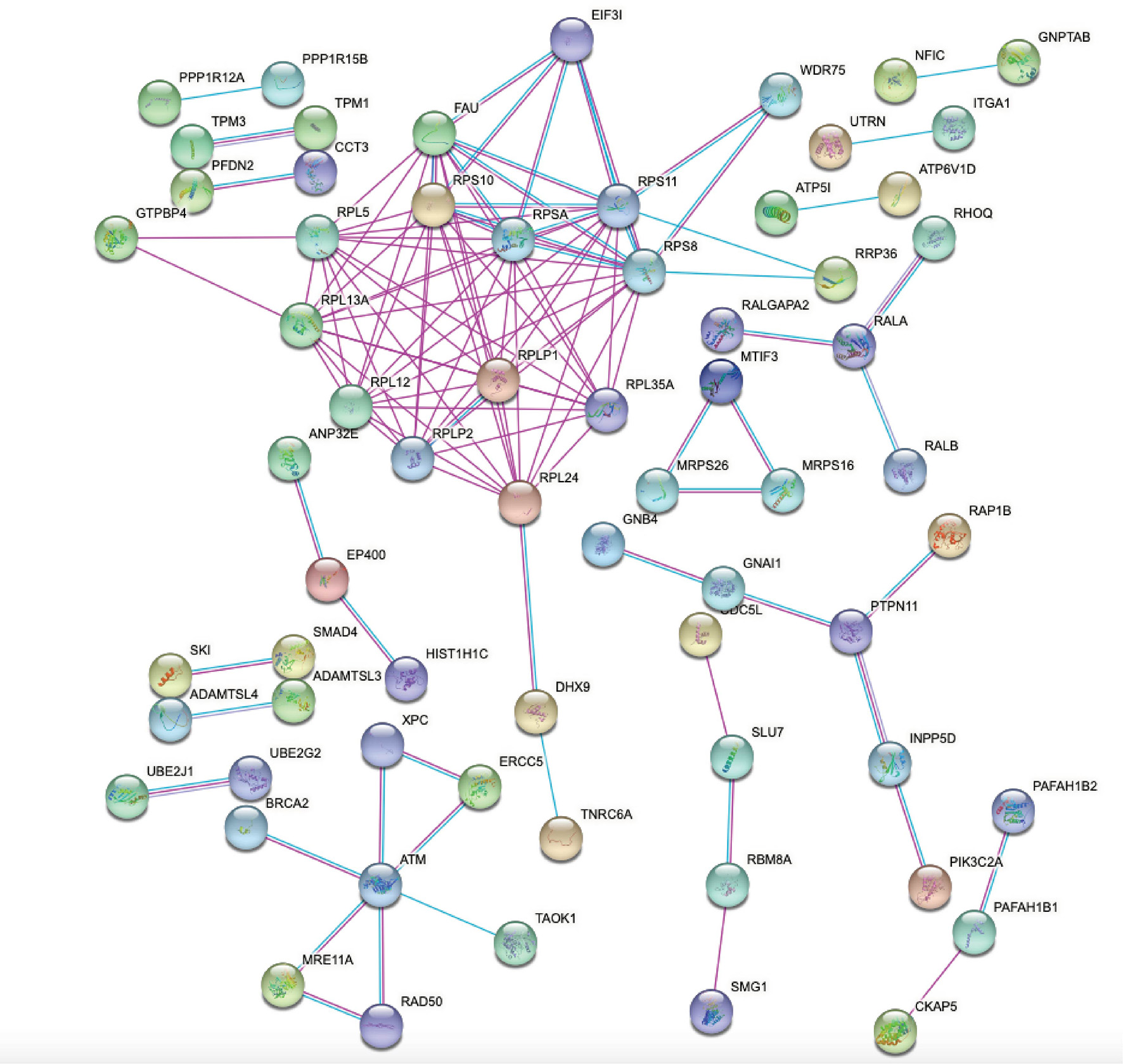

Supplement: Supplementary Figure 1 — Network analysis of the common gene set (n = 244) in the placenta. Interactome of gene–gene interactions representing joint contribution to a shared function. Protein interaction network analysis indicates the genes belonging to the eukaryotic translation initiation factor 2 (eIF2), mammalian target of rapamycin (mTOR), and interleukin-6 (IL-6) Signaling pathways playing a central role in the interactome of placenta that are associated with cerebral white matter damage. All 244 genes were used as input for STRING analysis and a network of 64 proteins was built (Supplementary Table 1a). Shown are the details of protein-protein interactions based on highest confidence (0.9) evidence from experimental protein-protein interaction (purple lines) and curated (blue lines) databases. Proteins are indicated by nodes labeled with the encoding gene symbol. Nodes of disconnected proteins were hidden. The network is enriched in interactions (p = 4.2E-10). [file Image_1.JPEG]

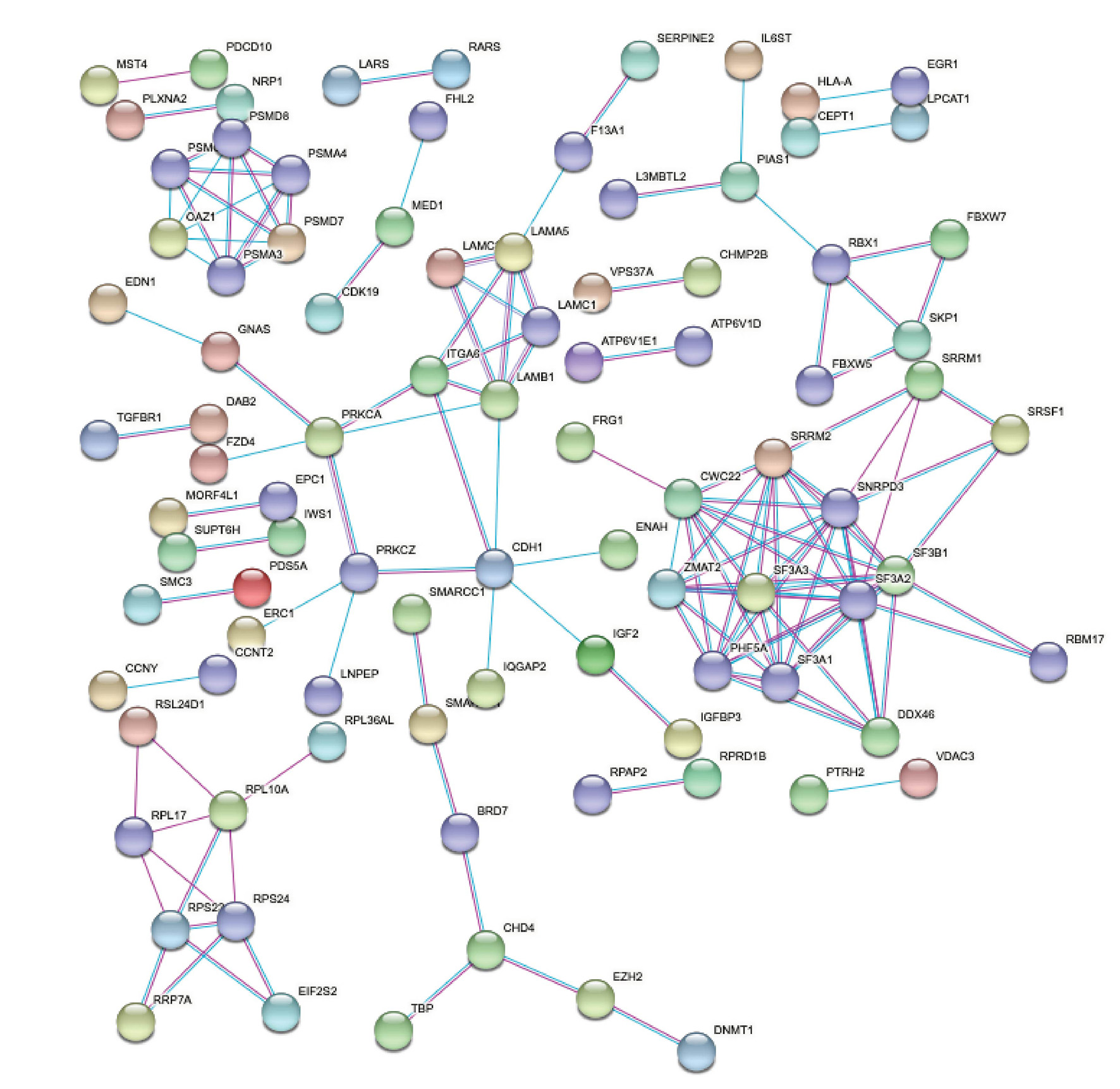

Supplement: Supplementary Figure 2 — Network analysis of the echolucency unique genes (n = 381) in the placenta. Interactome of gene–gene interactions representing joint contribution to a shared function. Protein interaction network analysis indicates the genes belonging to the growth hormone pathway playing a central role in the interactome of placenta that are associated with cerebral white matter damage. All 381 genes were used as input for STRING analysis and a network of 106 proteins was built (Supplementary Table 1a). Shown are the details of protein-protein interactions based on highest confidence (0.9) evidence from experimental protein-protein interaction (purple lines) and curated (blue lines) databases. Proteins are indicated by nodes labeled with the encoding gene symbol. Nodes of disconnected proteins were hidden. The network is enriched in interactions (p = 0.00405). [file Image_2.JPEG]

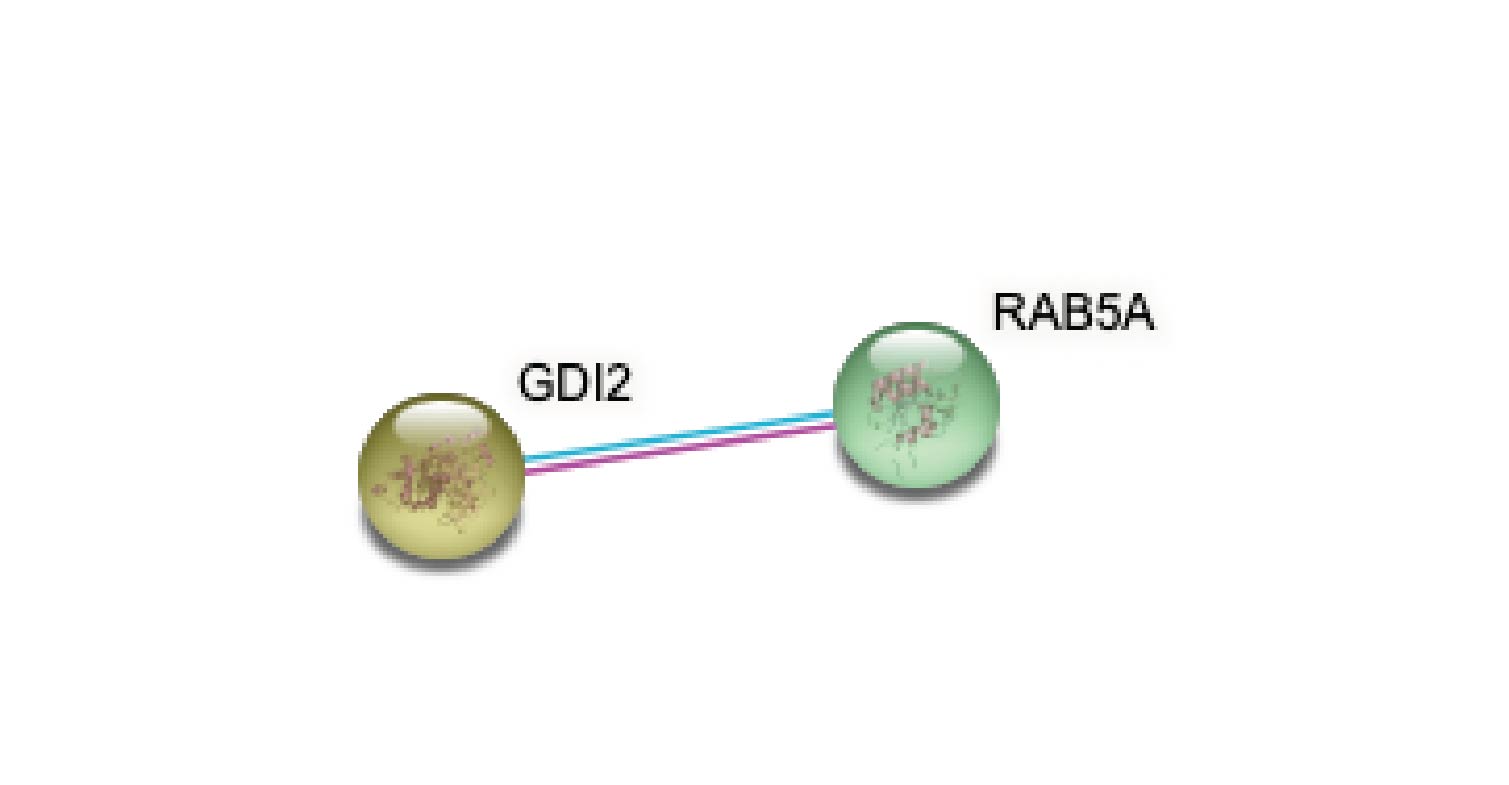

Supplement: Supplementary Figure 3 — Network analysis of the ventriculomegaly unique genes (n = 34) in the placenta. Interactome of gene–gene interactions representing joint contribution to a shared function. Protein interaction network analysis indicates the genes belonging to the clathrin-mediated endocytosis signaling pathway playing a central role in the interactome of placenta that are associated with cerebral white matter damage. All 381 genes were used as input for STRING analysis and a network of two proteins was built (Supplementary Table 1a). Shown are the details of protein-protein interactions based on highest confidence (0.9) evidence from experimental protein-protein interaction (purple lines) and curated (blue lines) databases. Proteins are indicated by nodes labeled with the encoding gene symbol. Nodes of disconnected proteins were hidden. The network is enriched in interactions (p = 0.569). [file Image_3.JPEG]
